# Decoding the RNA Regulatory Network in Medaka (*Oryzias latipes*) Spermatogenesis: Insights from a Germ Cell Reprogramming Model

**DOI:** 10.3390/ani16030389

**Published:** 2026-01-26

**Authors:** Manying Zhou, Jingjie Liang, Ke Lu, Yuewen Jiang, Yan Huang, Tiansheng Chen

**Affiliations:** 1 State Key Laboratory of Mariculture Breeding, Key Laboratory of Healthy Mariculture for the East China Sea, Ministry of Agriculture and Rural Affairs, Fisheries College, Jimei University, Xiamen 361021, China; 202311710011@jmu.edu.cn (M.Z.); 202261000039@jmu.edu.cn (J.L.); 202161000121@jmu.edu.cn (Y.H.); 2 College of Fisheries, Huazhong Agricultural University, Wuhan 430070, China; luke@mail.hzau.edu.cn (K.L.); jyuewen@webmail.hzau.edu.cn (Y.J.)

**Keywords:** spermatogenesis, germ cells, endogenous RNA (ceRNA), circular RNA (circRNA), microRNA (miRNA)

## Abstract

Spermatogenesis is a sophisticated process coordinated by germ cells and the somatic microenvironment. Circular RNAs (circRNAs), key components of competitive endogenous RNA (ceRNA) networks, form intricate post-transcriptional regulatory systems by sequestering microRNAs (miRNAs). However, the specific functions of these networks in spermatogenesis, particularly regarding the cell-intrinsic regulatory programs of germ cells, remain poorly understood. To address this, we utilized a unique *foxl3* mutant model in medaka (*Oryzias latipes*), in which XX female germ cells spontaneously transdifferentiate into functional sperm within the ovarian somatic environment. This model enables the functional enrichment of core spermatogenic programs largely independent of male-specific somatic cues. Through whole-transcriptome sequencing and bioinformatic analysis, we identified 58 key circRNAs, 27 core miRNAs, and 2965 mRNAs, and constructed a candidate ceRNA regulatory network mediated by six circRNAs. Under genetically consistent conditions, this study elucidated a putative ceRNA network directly involved in the germ cell-dominant initiation of spermatogenesis, suggesting an essential role of these networks in germ cell fate determination. These findings provide new insights into the regulatory mechanisms of teleost spermatogenesis and offer valuable molecular targets for advancing reproductive medicine and improving breeding efficiency in aquaculture.

## Simple Summary

Spermatogenesis—the biological process of sperm production—is essential for vertebrate reproduction and is typically thought to require a male-specific environment. However, the extent to which germ cells (reproductive cells) can coordinate the complex stages of sperm development through their intrinsic regulatory programs remains poorly understood. In this study, we utilized a unique Japanese medaka fish model where, due to the loss of the *foxl3* gene, female germ cells initiate and complete functional sperm production within an ovarian environment. This allowed us to enrich for the core genetic program of spermatogenesis largely independent of external male-specific somatic signals. By analyzing the complete RNA regulatory landscape, we identified a candidate network of molecules that may act as internal controllers for sperm development. We discovered that a specific group of RNAs likely functions as a central regulatory hub, associated with the pathways necessary for sperm maturation and structural formation. These findings provide a new understanding of the relative functional independence of germ cells during spermatogenesis. This research offers valuable candidate molecular targets for advancing reproductive medicine and improving breeding efficiency in aquaculture.

## 1. Introduction

Spermatogenesis relies on the coordination between the intrinsic developmental programs of germ cells and signals from the somatic microenvironment in the testis [1,2,3]. While the somatic signals have been extensively studied, the cell-autonomous regulatory network driving germ cell differentiation into sperm remains poorly understood. Understanding this core program is crucial for decoding the regulatory mechanisms of spermatogenesis.

Recent research on non-coding RNAs has shifted focus from classical signaling pathways to post-transcriptional regulatory networks. The competitive endogenous RNA (ceRNA) mechanism, where RNA molecules cross-talk through shared microRNA (miRNA) binding sites, provides a new perspective on gene regulation [4,5]. Circular RNAs (circRNAs), with their closed-loop structure and abundance of miRNA binding sites, are critical ceRNA carriers or “molecular sponges” [6,7,8,9]. While miRNA regulation in spermatogenesis is well-established, the role of circRNAs remains unclear. Increasing evidence suggests that circRNAs function as ceRNAs and also bind RNA-binding proteins or encode small peptides, with dynamic expression patterns in testicular tissue and spermatogenesis, highlighting their regulatory importance [10,11,12,13,14,15,16,17,18]. Thus, analyzing the ceRNA network mediated by circRNAs in spermatogenesis, especially in the cell-autonomous regulation of germ cells, is a key scientific challenge.

However, in organisms like fish, where sex determination is more plastic and influenced by environmental and epigenetic factors [19], separating the intrinsic germ cell program from external influences is difficult. Traditional studies of male and female individuals often confuse the contributions of genetic sex, gonadal somatic cell composition, and germ cell development, making it hard to highlight the core events associated with germ cell differentiation.

This study uses the unique *foxl3* mutant model in medaka (*Oryzias latipes*), where *foxl3* mutation leads to the spontaneous transdifferentiation of XX female germ cells into functional sperm within the ovarian somatic environment [20]. This model allows the functional enrichment of the spermatogenic program largely independent of the male-specific somatic microenvironment, enabling direct comparison of germ cells supported by ovarian somatic cells with those initiating spermatogenesis, all under the same XX genetic background. This design enables the identification of candidate circRNAs, miRNAs, and mRNAs potentially involved in the “masculinization” of germ cells while minimizing confounding somatic interference.

By comparing whole-transcriptome data from wild-type medaka testis, ovary, and *foxl3* mutant gonads, this study aims to map a ceRNA network associated with the core spermatogenesis program. This will provide new insights into germ cell fate transformation and help clarify the balance between cell-intrinsic developmental programs and external environmental influences, offering a foundation for improving aquaculture breeding technologies.

## 2. Methods and Materials

### 2.1. Experimental Animals

The Japanese medaka (HdrR strain) was maintained in a recirculating water system at 26–28 °C on a 14-h light/10-h dark cycle. The fish were fed newly hatched Artemia three times daily to ensure proper growth and reproduction. Embryos were collected from breeding pairs, placed in Petri dishes with 1× embryo incubation solution, and incubated at 28 °C under light.

The *foxl3* mutant was generated using CRISPR/Cas9 [21,22]. A guide RNA (gRNA) targeting the *foxl3* gene (GeneID: 101162366) was designed using the ChopChop web tool (https://chopchop.cbu.uib.no/). The target sequence was 5′-gaagccgccctattcgtacgtgg-3′. A mixture of 50 ng/μL gRNA and 300 ng/μL Cas9 protein was injected into single-cell stage embryos. Three days later, embryo DNA was extracted using alkaline lysis, and gRNA cleavage activity was verified by PCR and Sanger sequencing. After successful verification, the F0 generation was raised to adulthood and crossed with wild-type medaka to produce F1 offspring. F1 medaka with the same mutation were paired to generate F2 homozygous mutants, which exhibited intersex gonads in females and were used for subsequent analysis.

All experimental procedures were approved by the Institutional Animal Care and Use Committee of Jimei University (JMU202203009).

### 2.2. Tissue Sectioning and HE Staining

Wild-type medaka testis (WT_T), wild-type ovary (WT_O), and *foxl3* knockout intersex ovary (*foxl3*_O) tissues were fixed overnight in 4% paraformaldehyde (Servicebio, Wuhan, China) at 4 °C. After PBS washing, tissues were dehydrated in 20% and 30% sucrose solutions to prevent ice crystal formation, embedded in OCT compound (Sakura Finetek, Torrance, CA, USA), and frozen in liquid nitrogen. Using a pre-cooled cryostat, 8 μm sections were cut, mounted on adhesive slides, and stored at −20 °C. Sections were rehydrated, stained with hematoxylin for 3 min, rinsed with running water, differentiated with 1% hydrochloric acid ethanol, and blued with 0.6% ammonium hydroxide. After counterstaining with 0.5% eosin for 1 min, sections were dehydrated in graded ethanol, cleared in xylene, and mounted with neutral gum. Images were captured using an Olympus BX53 microscope (Olympus, Tokyo, Japan).

### 2.3. Total RNA Extraction and Quality Assessment

Total RNA was extracted from WT_T, WT_O, and *foxl3*_O tissues using TRIzol reagent (Thermo Fisher Scientific, Carlsbad, CA, USA) (three biological replicates per group; nine samples in total). RNA integrity was assessed by 1% agarose gel electrophoresis. RNA concentration and purity (A260/A280 and A260/A230 ratios) were measured using a Nanodrop 2000 spectrophotometer (Thermo Scientific, Wilmington, DE, USA). The RNA integrity number (RIN) was evaluated with an Agilent 2100 Bioanalyzer (Agilent Technologies, Santa Clara, CA, USA), and only samples with a RIN > 7.0 were used for RNA-Seq and small RNA-Seq library preparation.

### 2.4. RNA-Seq Analysis

Libraries for transcriptome sequencing were constructed using a strand-specific protocol. Ribosomal RNA was depleted using the Ribo-Zero™ rRNA Removal Kit (Illumina, San Diego, CA, USA). The RNA was fragmented (250–300 bp), reverse-transcribed into cDNA, and subjected to end repair, A-tailing, and adapter ligation. cDNA fragments (~200 bp) were selected using AMPure XP beads (Beckman Coulter, Brea, CA, USA) and amplified by PCR. Libraries were quantified using a Qubit 2.0 Fluorometer (Thermo Fisher Scientific, Waltham, MA, USA), sized using an Agilent 2100 Bioanalyzer (Agilent Technologies, Santa Clara, CA, USA), and their concentration confirmed by qRT-PCR (>3 nM). Paired-end sequencing (2 × 150 bp) was performed on an Illumina NovaSeq 6000 platform. Raw reads were processed with fastp (v0.23.2), aligned to the medaka reference genome (ASM223467v1) using HISAT2 (v2.2.1), and assembled using StringTie (v1.3.4). circRNAs were identified from unmapped reads by extracting 20-nt anchor sequences, realigning to the genome using Bowtie2 (v2.2.8), and detecting back-splice junctions with Find_circ and CIRI. Only circRNAs identified by both tools and supported by ≥2 reads were retained for further analysis.

### 2.5. Small RNA-Seq Analysis

Small RNA libraries were prepared from 3 μg of total RNA per sample using the Small RNA Sample Pre Kit, which ligates adapters to small RNAs. After reverse transcription and PCR amplification, DNA fragments (~140–160 bp) were size-selected by PAGE gel extraction. Libraries were quantified (Qubit 2.0), sized (Agilent 2100), and validated by qPCR (effective concentration > 2 nM) before sequencing. Single-end sequencing (50 bp reads) was performed on an Illumina NovaSeq 6000 platform. Raw data were filtered using fastp, and clean reads were aligned to the medaka genome (ASM223467v1) using Bowtie (v2.1.1) with zero mismatches. Known miRNAs were identified by comparison with the miRBase database (release 22), and potential target genes were predicted using miRanda (v2.2.1) and RNAhybrid (v2.1.2).

### 2.6. Quantification and Principal Component Analysis (PCA)

Expression levels were quantified using normalized metrics: FPKM (Fragments Per Kilobase of exon model per Million mapped fragments) for mRNAs, and TPM (Transcripts Per Million) for miRNAs and circRNAs. PCA was performed on log2-transformed expression matrices (log2(FPKM+1) for mRNAs; log2(TPM+1) for miRNAs and circRNAs) using the prcomp function in R (v4.2.1) to assess transcriptomic variance and sample clustering.

### 2.7. Differential Expression (DE) and Target Candidate Filtering

DE analysis was performed separately for mRNAs, circRNAs, and miRNAs using raw read counts as input. For all comparisons with biological replicates (*n* ≥ 3 per group), analysis was conducted using DESeq2 (v2.0). Genes or transcripts with |log2(fold change)| > 1 and an adjusted *p*-value (*padj*) < 0.05 were defined as significantly differentially expressed. In cases where the *padj* threshold yielded too few results, a more lenient cutoff of *p*-value < 0.05 was applied.

To specifically identify RNA molecules associated with germ cell-driven spermatogenesis and minimize the potential “noise” from residual oocytes in the intersex *foxl3_O* gonads, we implemented a comparative filtering strategy. Specifically, transcripts (mRNAs, circRNAs, and miRNAs) were considered candidates only if they exhibited a consistent expression trend in both the WT_T vs. WT_O and *foxl3_O* vs. WT_O comparisons. By focusing on genes up-regulated in both functional testes and transdifferentiating ovaries relative to normal ovaries, we effectively enriched for the spermatogenic program while filtering out the baseline ovarian transcriptomic signatures.

### 2.8. Functional Enrichment Analysis

Gene Ontology (GO) enrichment analysis was performed on gene sets derived from RNAs showing consistent expression trends between WT_T and *foxl3*_O. Three categories were analyzed separately: (1) the coordinately expressed mRNAs themselves, (2) predicted target genes of the coordinately expressed miRNAs, and (3) host genes of the coordinately expressed circRNAs. Enrichment was assessed using the clusterProfiler package (v4.12.6) in R (v4.4.0), with terms having a *padj* < 0.05 considered significant.

### 2.9. ceRNA Network Construction

ceRNA network was constructed by integrating differentially expressed mRNAs, circRNAs, and miRNAs. The selection of the mRNAs within the network was based on three stringent criteria: (1) a consistent expression trend (both up-regulated or both down-regulated) in both *foxl3*_O and WT_T when compared to WT_O; (2) the presence of predicted binding sites for the identified core miRNAs; and (3) an expression profile negatively correlated with their targeting miRNAs. This dual-comparison approach (identifying shared regulatory signatures in both *foxl3*_O and WT_T) ensured that the core members were more likely associated with the transdifferentiating germ cells rather than the residual ovarian somatic background. The network was visualized and analyzed using Cytoscape (v3.10.0).

### 2.10. Experimental Validation of circRNAs

Candidate circRNAs were validated through RNase R resistance assay and junction-specific PCR. Total RNA (1 µg) was treated with RNase R (2 U) using the GSPure RNase R Kit (Geneseed Biotech Co., Ltd., Guangzhou, China) to enrich circRNAs by digesting linear RNAs. Subsequently, cDNA was synthesized from both treated and untreated RNA. For each circRNA, divergent primers spanning the predicted back-splice junction and convergent primers (control) were used for PCR amplification. All primer sequences are listed in Supplementary Table S1. PCR was performed using 2× NG PCR MasterMix (HLingene Biotech Co., Ltd., Shanghai, China) (35 cycles; annealing at 60 °C). PCR products were gel-purified using the Omega Gel Extraction Kit (Omega Bio-tek, Inc., Norcross, GA, USA) and confirmed by Sanger sequencing to verify the precise back-splice junction.

### 2.11. qRT-PCR Validation

To validate the sequencing data, the expression levels of 6 DE circRNAs, 3 DE mRNAs, and 3 DE miRNAs were analyzed by quantitative real-time PCR (qRT-PCR). Total RNA was reverse transcribed using HiScript IV RT SuperMix for qPCR (Vazyme, Nanjing, China) for circRNAs/mRNAs, and the miRNA 1st Strand cDNA Synthesis Kit (Vazyme) for miRNAs. qPCR was performed using ChamQ Universal SYBR qPCR Master Mix (Vazyme). *β-actin* and *U6* served as internal controls for circRNAs/mRNAs and miRNAs, respectively. Primer sequences are listed in Supplementary Table S1. Relative expression was calculated using the 2^−ΔΔCt^ method [23] and compared with sequencing results.

### 2.12. Statistical Analysis

All quantitative data are expressed as mean ± standard deviation (SD). Statistical analyses were conducted using GraphPad Prism 9.3 (GraphPad Software, San Diego, CA, USA). Differences between two groups were assessed using unpaired two-tailed Student’s *t*-tests, with *p* < 0.05 considered statistically significant.

## 3. Results

### 3.1. Sample Characterization

**Figure 1:**
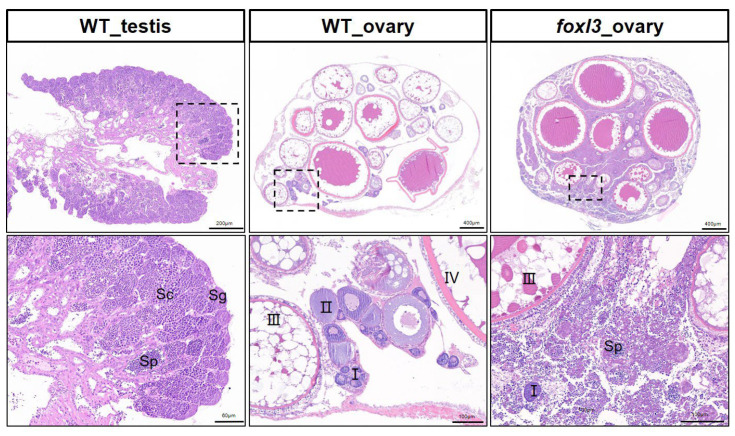
**HE-stained gonadal sections from sexually mature wild-type and *foxl3* mutant medaka.** The bottom row shows higher magnification views of the areas indicated by dashed boxes in the corresponding top panels. I–IV indicate the different developmental stages of oocytes. Sg: spermatogonia; Sc: spermatocytes; Sp: spermatozoa.

To delineate candidate ceRNA networks in spermatogenesis, we compared gonadal tissues from medaka: wild-type testis (WT_T), wild-type ovary (WT_O), and *foxl3* mutant ovary (*foxl3*_O). Histological examination confirmed the established mutant phenotype, which is consistent with previous reports [20]: while *foxl3*_O tissue retained oocytes, it also exhibited clear signs of spermatogenesis with sperm present (Figure 1), indicating the partial transdifferentiation of germ cells. Thus, comparing WT_O and *foxl3*_O allows for the identification of transcriptomic changes correlated with the acquisition of male germ cell fate and spermatogenesis within an XX context, largely independent of male-specific somatic cues.

### 3.2. Sequencing Data Overview

RNA-seq and sRNA-seq were performed on these gonadal tissues. RNA-seq generated approximately 895 million raw reads, and after quality control, all three groups (WT_T, WT_O, *foxl3*_O) yielded over 91 million high-quality clean reads, with unique mapping rates exceeding 83% (Supplementary Table S2). sRNA-seq produced about 167 million raw reads, with clean reads ranging from 17.3 to 19.3 million and an overall mapping rate exceeding 90% (Supplementary Table S3). Known miRNAs accounted for a significant portion of the small RNA population, particularly in WT_O (34.02%) and *foxl3*_O (23.06%), confirming the efficient capture of miRNA expression profiles in the gonads (Supplementary Table S4). These high-quality transcriptomic data provide a reliable foundation for identifying candidate differentially expressed molecules and constructing a testable ceRNA regulatory framework.

### 3.3. Identification, Characterization, and Differential Expression Analysis of circRNAs

**Figure 2:**
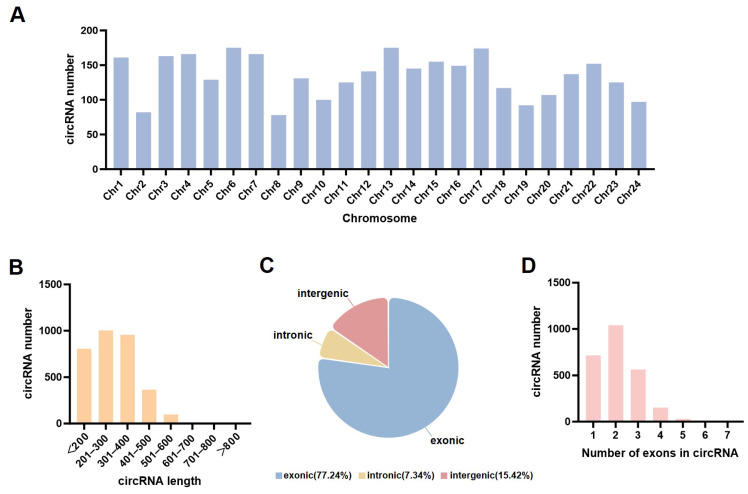
**Characterization and Classification of circRNAs.** (**A**) Distribution of circRNAs across chromosomes. (**B**) Length distribution of circRNAs. (**C**) Classification of circRNA types. (**D**) Number of exons within circRNAs.

A total of 3243 high-confidence circRNAs were identified across all samples through systematic screening of the whole transcriptome data. Genomic feature analysis (Figure 2A) showed that circRNAs are unevenly distributed across chromosomes, with lengths mainly in the 200–400 bp range (Figure 2B). Exonic circRNAs dominated (77.24%) (Figure 2C), with most formed by the cyclization of 1–3 exons (Figure 2D).

**Figure 3:**
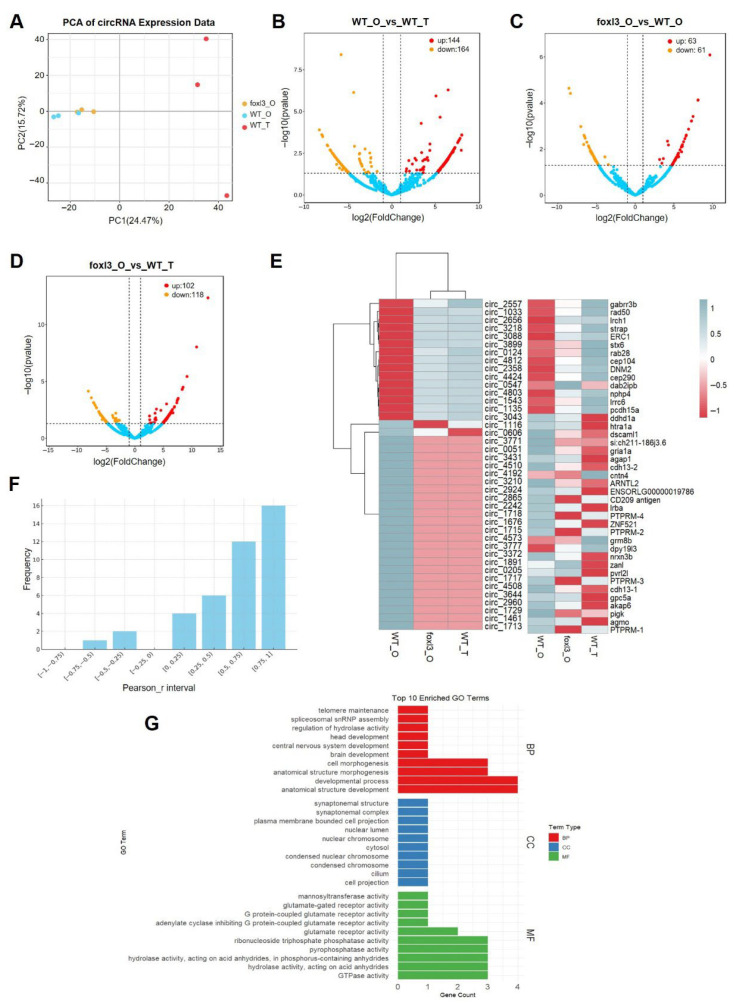
**CircRNA Expression Profiling and Differential Expression Analysis Across Gonadal Samples.** (**A**) PCA of circRNA expression levels in 9 gonadal samples. (**B**–**D**) Volcano plots of differentially expressed (DE) circRNAs between WT_O vs. WT_T (**B**), *foxl3*_O vs. WT_O (**C**), and *foxl3*_O vs. WT_T (**D**). Red and orange dots represent significantly up-regulated and down-regulated circRNAs, respectively, while blue dots represent non-significantly expressed circRNAs. (**E**) Heatmap of selected circRNAs and their host gene expression profiles in WT_T, WT_O, and *foxl3*_O. (**F**) Frequency plot showing the correlation between selected circRNAs and their host genes. (**G**) GO analysis of selected host genes of DE circRNAs.

PCA revealed distinct circRNA expression profiles across gonadal types. As shown in Figure 3A, PC1 (24.47%) separated wild-type testis (WT_T) from both ovarian tissues (WT_O, *foxl3*_O), reflecting fundamental gonadal differences. PC2 (15.72%) clustered *foxl3*_O with WT_O, distinct from WT_T, suggesting that, despite sperm presence in *foxl3*_O, its global circRNA profile remains substantially influenced by the ovarian somatic environment and residual oocytes.

Pairwise comparisons identified differentially expressed circRNAs (Figure 3B–D). In the *foxl3*_O vs. WT_O comparison, 124 circRNAs were identified, representing candidate molecules potentially linked to the masculinization of germ cells. For reference, 308 circRNAs were differentially expressed in WT_T vs. WT_O, and 220 circRNAs in *foxl3*_O vs. WT_T.

To enrich for circRNAs most relevant to spermatogenesis and minimize background interference from the ovarian niche, we selected those with consistent expression trends in both *foxl3*_O and WT_T (upregulated or downregulated in both comparisons relative to WT_O), leading to the identification of 58 candidate circRNAs. These circRNAs were mainly derived from coding genes (42 genes), with 37 originating from exons (Figure 3E). Correlation analysis showed most circRNAs were positively correlated with the expression of their host genes (Figure 3F), while three circRNAs (circ_3043-*ddhd1a*, circ_4573-*grm8b*, circ_3777-*dpy19l3*) were negatively correlated, suggesting they may have independent regulatory roles.

GO functional enrichment analysis of the host genes of these circRNAs (Figure 3G) revealed significant enrichment in biological processes such as “anatomical structure development,” “cell morphogenesis”; in cellular components like “cilium” and “nuclear chromosomes”; and in molecular functions like “hydrolase activity” and “glutamate receptor activity”, providing further evidence of their putative roles in germ cell differentiation.

### 3.4. miRNA Identification and Differential Expression Analysis

**Figure 4:**
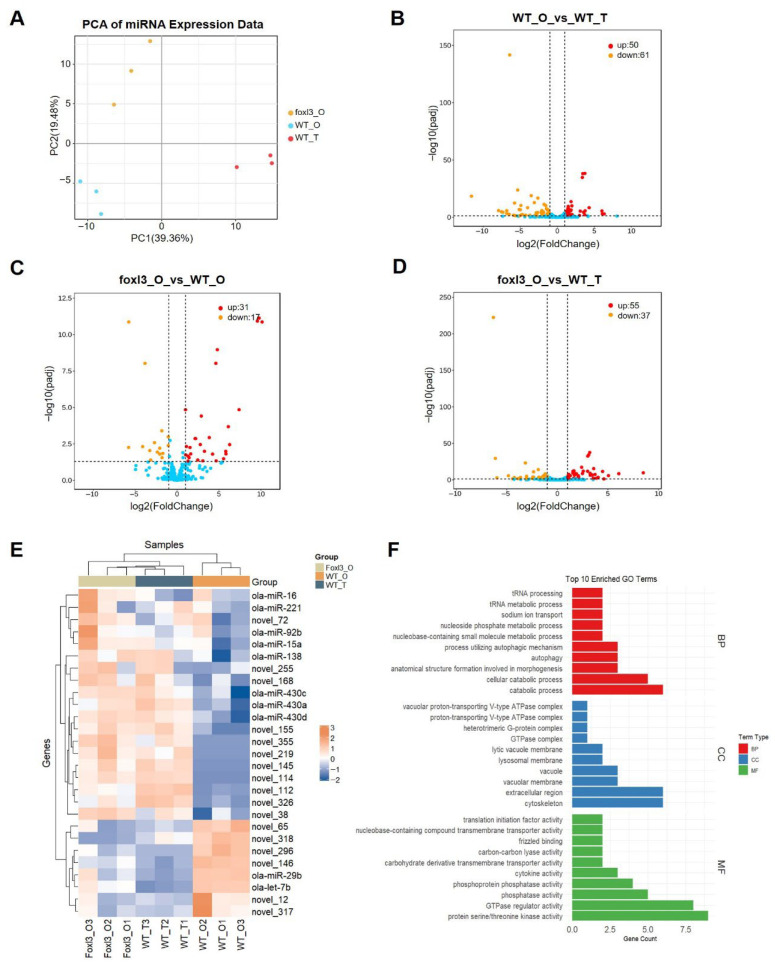
**Profiling and Differential Expression Analysis of miRNAs Across Gonadal Samples.** (**A**) PCA of miRNA expression levels in 9 gonadal samples. (**B**–**D**) Volcano plots of differentially expressed (DE) miRNAs between WT_O vs. WT_T (**B**), *foxl3*_O vs. WT_O (**C**), and *foxl3*_O vs. WT_T (**D**). Red and orange dots represent significantly up-regulated and down-regulated miRNAs, respectively, while blue dots represent non-significantly expressed miRNAs. (**E**) Heatmap of selected miRNA expression profiles in WT_T, WT_O, and foxl3_O. (**F**) GO analysis of target genes of DE miRNAs.

Analysis of small RNA sequencing data identified 275 miRNAs across all gonadal samples (139 known and 136 newly predicted). PCA revealed that PC1 (39.36%) and PC2 (19.48%) together accounted for 58.84% of the variation. The PCA plot (Figure 4A) showed that WT_T was distinct from the ovarian samples (WT_O and *foxl3*_O) along PC1, while *foxl3*_O was separated from WT_O along PC2, indicating significant reprogramming of the miRNA expression profile in the *foxl3* mutant gonads during germ cell transdifferentiation.

Pairwise comparisons identified differentially expressed miRNAs (Figure 4B–D). In the *foxl3*_O vs. WT_O comparison, 48 miRNAs were differentially expressed, with 31 upregulated in *foxl3*_O, suggesting their potential involvement in germ cell masculinization. For reference, 111 miRNAs were differentially expressed in the WT_T vs. WT_O comparison, and 92 in *foxl3*_O vs. WT_T.

To identify miRNAs most relevant to the spermatogenic program while filtering out the ovarian background, we selected those with consistent expression trends in both *foxl3*_O and WT_T, leading to the identification of 27 core candidate miRNAs (Figure 4E). Based on their expression patterns in WT_T, these miRNAs were classified into two groups: 19 highly expressed in the testis, potentially promoting spermatogenesis, and 8 with low expression, putatively acting as inhibitors.

Target gene prediction and functional enrichment analysis of these miRNAs (Figure 4F) revealed significant enrichment in biological processes such as tRNA processing, autophagy, cell catabolic processes, and morphogenesis. In cellular components, they were enriched in vacuoles, lysosomes, cytoskeleton, and other structures, while in molecular functions, they were enriched in protein serine/threonine kinase activity, GTPase regulator activity, and phosphatase activity, among other enzyme-related functions.

### 3.5. mRNA Identification and Differential Expression Analysis

**Figure 5:**
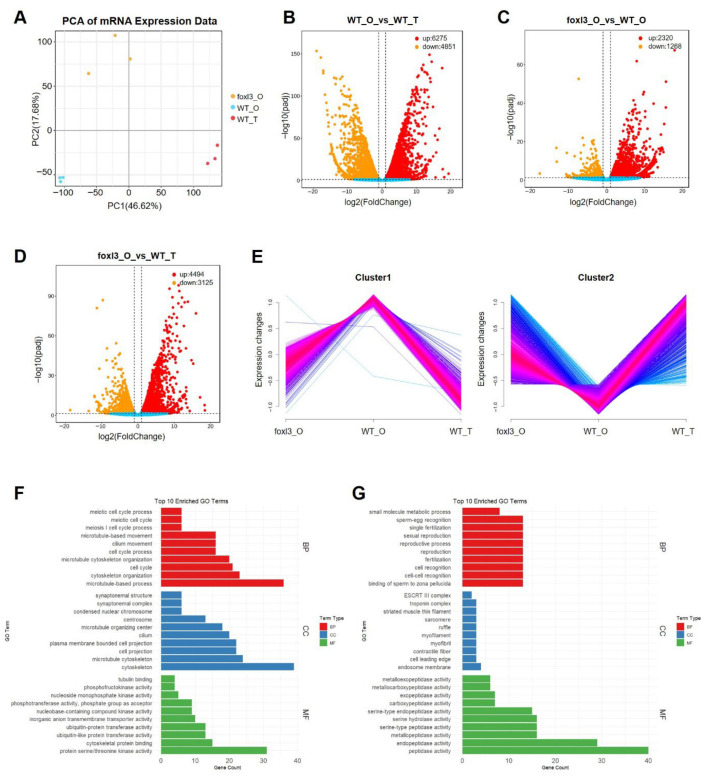
**Differential Expression and Functional Analysis of mRNAs Across Gonadal Samples.** (**A**) PCA of mRNA expression levels in 9 gonadal samples. (**B**–**D**) Volcano plots of differentially expressed (DE) mRNAs between WT_O vs. WT_T (**B**), *foxl3*_O vs. WT_O (**C**), and *foxl3*_O vs. WT_T (**D**). Red and orange dots represent significantly up-regulated and down-regulated mRNAs, respectively, while blue dots represent non-significantly expressed mRNAs. (**E**) Clustering of gene expression changes across gonadal samples, visualized as line plots. Expression changes are indicated by color gradients, with blue representing low expression and red representing high expression. (**F**) GO analysis of DE genes upregulated in testis. (**G**) GO analysis of DE genes downregulated in testis.

A total of 39,399 transcripts corresponding to 24,321 genes were identified, including 717 newly predicted genes. PCA revealed that PC1 (46.62%) clearly separated WT_T from the two ovarian samples, with PC2 (17.68%) further distinguishing the *foxl3*_O from the WT_O (Figure 5A), indicating a distinct transcriptomic shift in the mutant gonads toward a male-like state despite the ovarian somatic context. DE analysis showed 11,126 DE genes between WT_T and WT_O, with 6275 upregulated in testis and 4851 upregulated in ovary. Between *foxl3*_O and WT_O, 3588 DE genes were identified, with 2320 upregulated in the mutant. Additionally, 7619 DE genes were found between t *foxl3*_O and WT_T, with 4494 upregulated in the mutant (Figure 5B–D).

To identify core mRNAs specifically associated with the spermatogenic program, we selected genes with consistent expression patterns in both *foxl3*_O and WT_T, identifying 2965 candidate genes. These were classified into two groups: 1846 upregulated in both and 1119 downregulated in both (Figure 5E, Supplementary Table S5). Functional enrichment analysis revealed that genes highly expressed in the testis were significantly enriched in essential spermatogenic processes like the cell cycle, meiosis, and microtubule organization, and in cellular components related to microtubules and the cytoskeleton (Figure 5F). Genes with low expression in the testis were enriched in processes related to reproduction and gamete recognition, localized to cell fronts and endosomal membranes, and exhibited peptidase and serine-type endopeptidase activities (Figure 5G). The enrichment of these conserved spermatogenic pathways further supports the validity of our comparative filtering strategy in capturing germ cell-intrinsic regulatory events.

### 3.6. Construction of the Candidate ceRNA Regulatory Network

**Figure 6:**
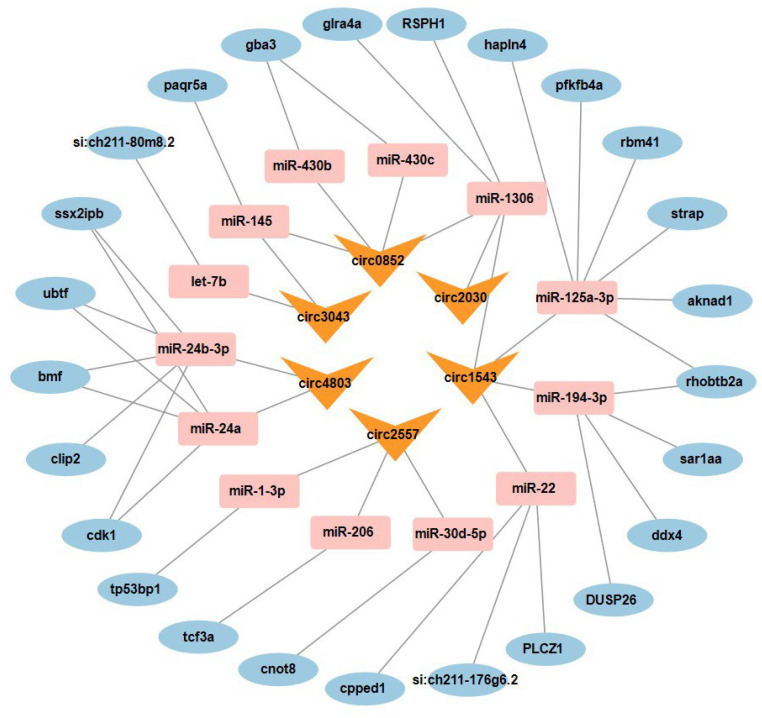
**ceRNA network analysis of 6 spermatogenesis-associated circRNAs using Cytoscape.** CircRNAs are represented in yellow, miRNAs in red, and mRNAs in blue.

Six circRNAs with consistent expression trends in both WT_T and *foxl3*_O were prioritized to explore their candidate regulatory mechanisms. Bioinformatics analysis identified miRNAs that could bind to these circRNAs, and subsequent screening identified their downstream target mRNAs. To ensure the network was specific to the spermatogenic program and minimized ovarian background noise, target genes were strictly selected from the 2965 candidates previously identified as having consistent expression patterns in both *foxl3_O* and WT_T, and only those showing expression trends opposite to the miRNAs were retained. Based on these stringent criteria, a candidate ceRNA network comprising 6 circRNAs, 13 miRNAs, and 25 mRNAs was constructed (Figure 6), representing a testable regulatory framework for germ cell masculinization.

### 3.7. Validation of circRNA Circularization and Sequencing Data Reliability

**Figure 7:**
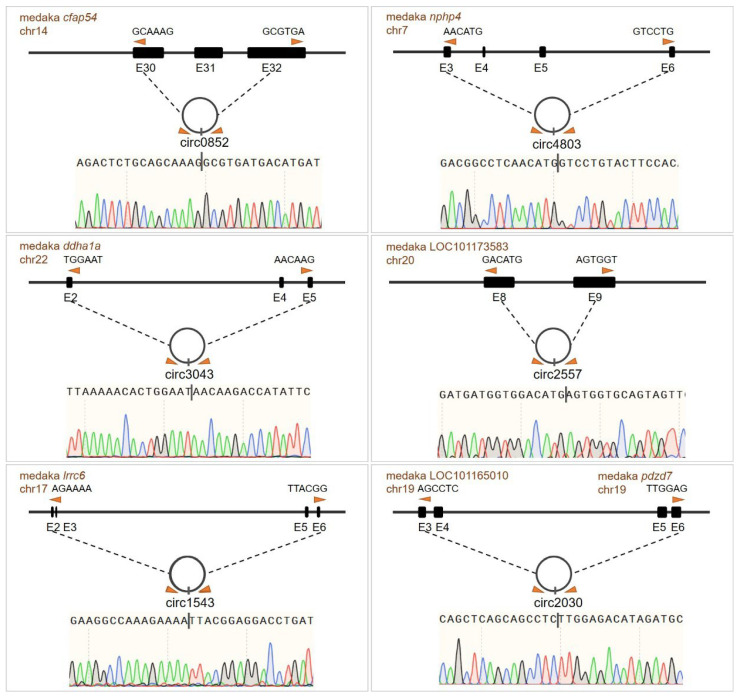
**Circular RNA sites within genes, validated by Sanger sequencing of back-splicing junctions.** The genomic loci of representative circRNAs are shown alongside their host genes. Orange triangles and dashed lines indicate the back-splicing sites forming the circular structures. For the Sanger sequencing chromatograms, the vertical black line denotes the back-splicing junction site. In the sequencing traces, the four colored peaks represent the four bases: green for A, red for T, blue for C, and black for G.

**Figure 8:**
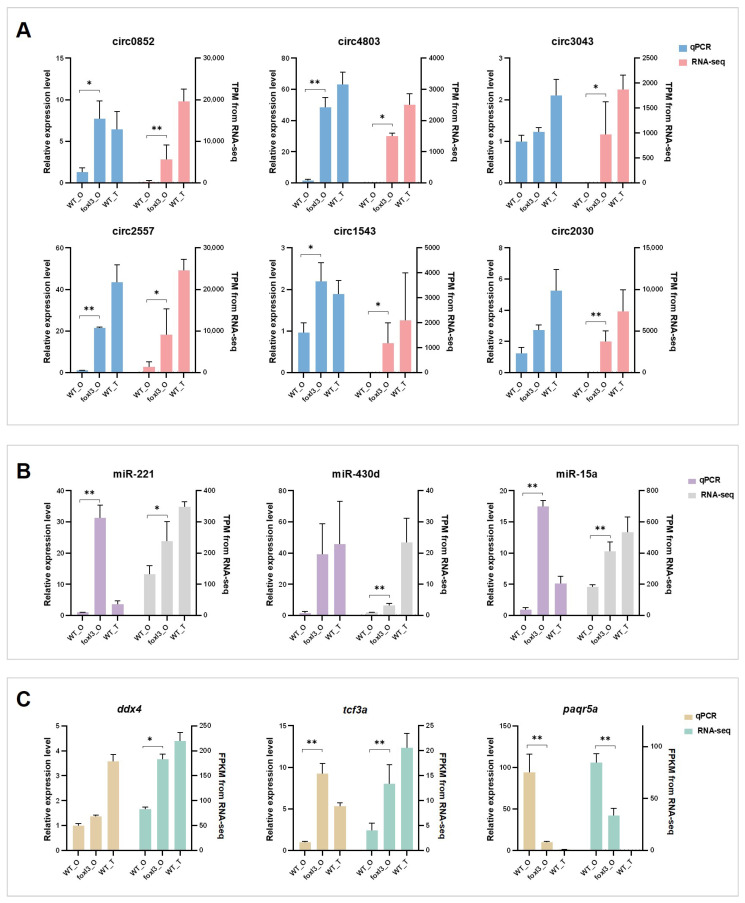
**qRT-PCR Validation of Gene Expression in Medaka Gonads.** (**A**) circRNAs, (**B**) miRNAs, and (**C**) mRNAs. qRT-PCR data were normalized using *β-actin* (for circRNAs and mRNAs) and *U6* snRNA (for miRNAs) as internal reference genes. Asterisks indicate significant differences between wild-type and mutant ovaries (* *p* < 0.05; ** *p* < 0.01).

To validate the reliability of the transcriptomic signatures, the six key circRNAs integrated into the ceRNA network were confirmed for circularization via RNase R resistance assays and junction-specific PCR followed by Sanger sequencing (Figure 7, Table 1). RT-qPCR was also used to measure the expression levels of randomly selected differentially expressed miRNAs and mRNAs in gonadal tissues. While absolute quantitative differences were observed between RT-qPCR and RNA-seq, the relative expression changes across groups were highly consistent (Figure 8). These results confirm the reliability of the sequencing data and provide experimental support for the inferred expression patterns of the core components within our candidate regulatory network.

## 4. Discussion

A key challenge in studying male germ cell differentiation is understanding the complex interactions between the cell-intrinsic regulatory program of germ cells and signals from the somatic microenvironment. Using the *foxl3* mutant medaka model, this study aimed to enrich for the spermatogenesis process largely independent of the male-specific somatic microenvironment. In this model, germ cells in XX individuals transdifferentiate into functional sperm in a somatic environment that retains female characteristics [20]. This phenomenon highlights the relative functional independence of germ cells in sex determination and provides a unique opportunity to reveal the molecular events associated with germ cell masculinization in an identical XX genetic background.

Based on this model, whole-transcriptome sequencing identified circRNAs, miRNAs, and mRNAs closely associated with spermatogenesis in medaka, leading to the construction of a candidate ceRNA regulatory network comprising 6 core circRNAs, 13 miRNAs, and 25 mRNAs. This network offers a putative molecular map for understanding the germ cell-dominant regulatory mechanisms of germ cell fate determination in teleost fish.

### 4.1. Hierarchical Regulation of Different RNA Molecules in Germ Cell Masculinization

Analysis of the overall expression patterns revealed the unique roles and distribution of different RNA molecules within the regulatory hierarchy. At the circRNA level, the overall expression profile of the *foxl3* mutant gonads closely resembled that of the wild-type ovary, a pattern supported by PCA. This suggests that the circRNAs that significantly change during the initiation of germ cell masculinization are relatively few in number. However, this “minimalist” feature indicates that these core circRNAs may act as key regulatory “switches” that trigger or amplify downstream pathways via mechanisms such as ceRNA. Notably, the expression of some circRNAs showed a negative correlation with their host genes, strongly suggesting that they may escape transcriptional control and possess independent, specific regulatory functions, playing an irreplaceable role in spermatogenesis.

In contrast to the “switch” function of circRNAs, miRNAs in the *foxl3* mutant gonads played a prominent “driver” role. Their expression reprogramming shifted from the WT_O profile towards that of the WT_T. For example, the miR-430 family, the largest miRNA cluster in teleosts, plays a critical role in early embryonic development and primordial germ cell migration and specialization [24,25,26,27,28,29,30]. In the rice field eel (*Monopterus albus*), a species capable of sex reversal, miR-430 family members are differentially expressed during the female-to-male transition, with miR-430b showing male-specific expression, implicating it in regulating sexual plasticity [31]. In medaka, the entire miR-430 family is highly expressed in males, supporting its conserved role in male sexual development. Notably, miR-430 was highly expressed in wild-type testis, low in ovary, and upregulated in *foxl3* mutant gonads, suggesting that germ cells can potentially initiate masculinization and transdifferentiation via miRNA-mediated programs largely independent of male-specific somatic niche. These findings highlight the conserved role of the miR-430 family in teleost sex determination, particularly in directing male germ cell fate.

As terminal effectors of the ceRNA network, the core mRNA set reveals the potential functional outcomes of germ cell transdifferentiation. Functional enrichment analysis showed clear transcriptional reprogramming: male reproductive programs were activated, with upregulated genes enriched in essential spermatogenic processes such as “meiosis,” “synaptonemal complex assembly,” and “cilia assembly”. In contrast, female-related programs were suppressed, with downregulated genes enriched in “egg-sperm recognition” and various “peptidase activities.” This bidirectional expression pattern forms a candidate molecular basis for the shift in germ cell fate, further validating the use of the *foxl3* model to enrich for germ cell-intrinsic programs largely independent of male-specific somatic cues.

### 4.2. ceRNA Network: From Global Mapping to Specific Regulatory Hypothesis

Building on mRNA functional patterns, we further explored the potential regulatory mechanisms upstream of these genes. To ensure the biological reliability of the candidate ceRNA network and to mitigate potential transcriptomic “noise” from the ovarian somatic background, we implemented a dual-stringent screening strategy. Specifically, miRNA-mRNA pairs with significant negative correlations were prioritized, provided the mRNAs belonged to the core spermatogenesis gene set—defined by consistent expression shifts in both *foxl3_O* and wild-type testis (WT_T) relative to wild-type ovary (WT_O). Since WT_T is devoid of oocyte components, the shared expression signatures between the mutant and functional testis strongly suggest that these core members are intrinsically associated with the transdifferentiating germ cells rather than the residual ovarian environment. Importantly, many of the selected candidates are well-characterized markers or essential factors for germ cell development and flagellar assembly—processes that are biologically absent in ovarian somatic compartments. The convergence of consistent expression across male-pathway tissues with their established roles in germ cell biology provides high confidence that the identified ceRNA module represents a germ cell-intrinsic regulatory program. This comparative approach refined a specific candidate ceRNA regulatory module from a large set of bioinformatics predictions.

Taking circ1543 as an example, we propose a testable regulatory hypothesis for further validation. This circRNA, formed by exons of its host gene *lrrc6*, which encodes LRRC6, a key factor in cilia/flagella formation [32,33], may act as a molecular sponge, sequestering miRNAs such as miR-125a-3p, miR-194-3p, miR-1306, and miR-22. These miRNAs are highly expressed in the ovary, suggesting they might inhibit masculinization. We hypothesize that during spermatogenesis, upregulation of circ1543 may buffer these ovarian miRNAs, relieving their translational repression of target genes like *ddx4* (*vasa*), a key germ cell marker essential for primordial germ cell migration and germ cell development [34,35], thus promoting spermatogenesis. This “multi-target” hypothesis demonstrates the ceRNA network’s potential efficiency in coordinating complex biological processes and provides a novel perspective on germ cell fate regulation. However, we explicitly acknowledge that the current network represents a bioinformatically inferred framework, and the lack of direct spatial evidence (e.g., in situ hybridization) for cellular localization remains a limitation. Future functional validation, including dual-luciferase reporter assays and cell-specific perturbations of key candidates, will be essential to definitively confirm these regulatory causalities and transition from hypothesis generation to mechanistic causality.

## 5. Conclusions

This study utilized the *foxl3* mutant medaka model to enrich for and analyze the germ cell-intrinsic regulatory signatures during spermatogenesis, proposing a candidate ceRNA regulatory framework. This work not only validates the unique value of this model in uncovering the relatively intrinsic regulatory mechanisms of germ cells but also demonstrates the potential ability of a limited set of hub molecules to coordinate and drive complex biological processes through network formation. Our findings offer new perspectives and valuable molecular resources for understanding the core principles of sex determination and differentiation in teleost fish. This work establishes a preliminary but logically robust regulatory framework, serving as a roadmap for future experimental validation of germ cell fate determination at the post-transcriptional level.

## Figures and Tables

**Table 1:** **Circularization Information of the Selected circRNAs**.

circRNA	Host Gene	Chromosome	Circularization Region
circ0852	*cfap54*	14	Exon 30, 31, 32 and intron between 31 and 32
circ4803	*nphp4*	7	Exon 3, 4, 5, 6
circ3043	*ddhd1a*	22	Exon 2, 4, 5
circ2557	*LOC101173583*	20	Exon 8, 9 and intervening intron
circ1543	*lrrc6*	17	Exon 2, 3, 5, 6
circ2030	*pdzd7/LOC101165010*	19	Exon 5, 6 of *pdzd7* and exon 3, 4 of *LOC101165010*

## Data Availability

The sequencing data are available in the Genome Sequence Archive (GSA) repository of the National Genomics Data Center. The data can be accessed via the following accession codes: CRA035279 and CRA034924.

## Supplementary Materials

The following supporting information can be downloaded at: https://www.mdpi.com/article/10.3390/ani16030389/s1, Table S1: Primers; Table S2: RNA-seq Quality Control and Mapping Statistics for Gonadal Samples; Table S3: Small RNA-seq (sRNA) Quality Control and Mapping Statistics for Gonadal Samples; Table S4: Classification of Small RNA Types and Mapping Statistics Across Gonadal Samples; Table S5: Core mRNAs candidates related to spermatogenesis.

## References

[1] Schulz R.W., de Franca L.R., Lareyre J.J., Le Gac F., Chiarini-Garcia H., Nobrega R.H., Miura T. (2010). Spermatogenesis in fish. Gen. Comp. Endocrinol..

[2] Griswold M.D. (2016). Spermatogenesis: The commitment to meiosis. Physiol. Rev..

[3] Liu W., Du L., Li J., He Y., Tang M. (2024). Microenvironment of spermatogonial stem cells: A key factor in the regulation of spermatogenesis. Stem Cell Res. Ther..

[4] Tay Y., Rinn J., Pandolfi P.P. (2014). The multilayered complexity of ceRNA crosstalk and competition. Nature.

[5] Thomson D.W., Dinger M.E. (2016). Endogenous microRNA sponges: Evidence and controversy. Nat. Rev. Genet..

[6] Hansen T.B., Jensen T.I., Clausen B.H., Bramsen J.B., Finsen B., Damgaard C.K., Kjems J. (2013). Natural RNA circles function as efficient microRNA sponges. Nature.

[7] Memczak S., Jens M., Elefsinioti A., Torti F., Krueger J., Rybak A., Maier L., Mackowiak S.D., Gregersen L.H., Munschauer M. (2013). Circular RNAs are a large class of animal RNAs with regulatory potency. Nature.

[8] Qu S., Yang X., Li X., Wang J., Gao Y., Shang R., Sun W., Dou K., Li H. (2015). Circular RNA: A new star of noncoding RNAs. Cancer Lett..

[9] Chen L.-L. (2016). The biogenesis and emerging roles of circular RNAs. Nat. Rev. Mol. Cell Biol..

[10] Capel B., Swain A., Nicolis S., Hacker A., Walter M., Koopman P., Goodfellow P., Lovell-Badge R. (1993). Circular transcripts of the testis-determining gene Sry in adult mouse testis. Cell.

[11] Dorsett D. (2020). Circular RNAs protect male fertility. Science.

[12] Cai Y.Q., Lei X.C., Chen Z., Mo Z.C. (2020). The roles of cirRNA in the development of germ cells. Acta Histochem..

[13] Gao L.Z., Chang S.H., Xia W.J., Wang X.L., Zhang C.W., Cheng L.P., Liu X., Chen L., Shi Q.H., Huang J. (2020). Circular RNAs from *BOULE* play conserved roles in protection against stress-induced fertility decline. Sci. Adv..

[14] Tang C., Xie Y.M., Yu T., Liu N., Wang Z.Q., Woolsey R.J., Tang Y.G., Zhang X.Z., Qin W.B., Zhang Y. (2020). m6A-dependent biogenesis of circular RNAs in male germ cells. Cell Res..

[15] Song Y., Chen M., Zhang Y., Li J., Liu B., Li N., Qiao M., Wang N., Cao Y., Lu S. (2022). Loss of circSRY reduces γH2AX level in germ cells and impairs mouse spermatogenesis. Life Sci. Alliance.

[16] Zhang S., Wang C., Wang Y., Zhang H., Xu C., Cheng Y.W., Yuan Y., Sha J.H., Guo X.J., Cui Y.Q. (2023). A novel protein encoded by circRsrc1 regulates mitochondrial ribosome assembly and translation during spermatogenesis. BMC Biol..

[17] Chen A.Q., Ji C.N., Li C.T., Brand-Saberi B., Zhang S.H. (2024). Multiple transcriptome analyses reveal mouse testis developmental dynamics. BMC Genom..

[18] Manfrevola F., Mosca N., Mele V.G., Chioccarelli T., Martinez G., Coutton C., Mattia M., Pezzullo M., Fasano S., Cobellis G. (2025). Deciphering the Contribution of Circular RNAs to Age-Related Decline in Sertoli Cell Survivor. Aging Cell.

[19] Nagahama Y., Chakraborty T., Paul-Prasanth B., Ohta K., Nakamura M. (2021). Sex determination, gonadal sex differentiation, and plasticity in vertebrate species. Physiol. Rev..

[20] Nishimura T., Sato T., Yamamoto Y., Watakabe I., Ohkawa Y., Suyama M., Kobayashi S., Tanaka M. (2015). foxl3 is a germ cell-intrinsic factor involved in sperm-egg fate decision in medaka. Science.

[21] Pan Q., Lu K., Luo J., Jiang Y., Xia B., Chen L., Wang M., Dai R., Chen T. (2023). Establishment of Japanese medaka (*Oryzias latipes*) mutants based on CRISPR/Cas9 system. J. Fish. China.

[22] Deng J., Huang Y., Liang J., Jiang Y., Chen T. (2024). Medaka (*Oryzias latipes*) *Dmrt3a* Is Involved in Male Fertility. Animals.

[23] Livak K.J., Schmittgen T.D. (2001). Analysis of relative gene expression data using real-time quantitative PCR and the 2(-Delta Delta C(T)) Method. Methods.

[24] Giraldez A.J., Mishima Y., Rihel J., Grocock R.J., Van Dongen S., Inoue K., Enright A.J., Schier A.F. (2006). Zebrafish MiR-430 promotes deadenylation and clearance of maternal mRNAs. Science.

[25] Mishima Y., Giraldez A.J., Takeda Y., Fujiwara T., Sakamoto H., Schier A.F., Inoue K. (2006). Differential regulation of germline mRNAs in soma and germ cells by zebrafish miR-430. Curr. Biol..

[26] (2010). Genomic organization and embryonic expression of miR-430 in medaka (*Oryzias latipes*): Insights into the post-transcriptional gene regulation in early development. Gene.

[27] Staton A.A. (2011). miRNA miR-430 Regulates Chemokine Signaling and Provides Robustness to Germ Cell Migration. Ph.D. Thesis.

[28] Staton A.A., Knaut H., Giraldez A.J. (2011). miRNA regulation of Sdf1 chemokine signaling provides genetic robustness to germ cell migration. Nat. Genet..

[29] Takacs C.M., Giraldez A.J. (2016). miR-430 regulates oriented cell division during neural tube development in zebrafish. Dev. Biol..

[30] Jimenez-Ruiz C.A., de la Herran R., Robles F., Navajas-Perez R., Cross I., Rebordinos L., Ruiz-Rejon C. (2023). miR-430 microRNA Family in Fishes: Molecular Characterization and Evolution. Animals.

[31] Gao Y., Guo W., Hu Q., Zou M., Tang R., Chi W., Li D. (2014). Characterization and Differential Expression Patterns of Conserved microRNAs and mRNAs in Three Genders of the Rice Field Eel (*Monopterus albus*). Sex. Dev..

[32] Kott E., Duquesnoy P., Copin B., Legendre M., Dastot-Le Moal F., Montantin G., Jeanson L., Tamalet A., Papon J.F., Siffroi J.P. (2012). Loss-of-Function Mutations in LRRC6, a Gene Essential for Proper Axonemal Assembly of Inner and Outer Dynein Arms, Cause Primary Ciliary Dyskinesia. Am. J. Human. Genet..

[33] Horani A., Ferkol T.W., Shoseyov D., Wasserman M.G., Oren Y.S., Kerem B., Amirav I., Cohen-Cymberknoh M., Dutcher S.K., Brody S.L. (2013). LRRC6 Mutation Causes Primary Ciliary Dyskinesia with Dynein Arm Defects. PLoS ONE.

[34] Shinomiya A., Tanaka M., Kobayashi T., Nagahama Y., Hamaguchi S. (2000). The vasa-like gene, olvas, identifies the migration path of primordial germ cells during embryonic body formation stage in the medaka, Oryzias latipes. Dev. Growth Differ..

[35] Aoki Y., Nagao I., Saito D., Ebe Y., Kinjo M., Tanaka M. (2008). Temporal and spatial localization of three germline-specific proteins in medaka. Dev. Dyn..

